# Acute Type A Aortic Dissection Surgery Performed by Aortic Specialists Improves 2-Year Outcomes

**DOI:** 10.1055/s-0039-1687904

**Published:** 2019-07-22

**Authors:** Syed Usman Bin Mahmood, Makoto Mori, Arnar Geirsson, John A. Elefteriades, Abeel A. Mangi

**Affiliations:** 1 Section of Cardiac Surgery, Department of Surgery, Yale University School of Medicine, New Haven, Connecticut

**Keywords:** ascending aortic surgery, acute Type A dissection, surgeon specialization

## Abstract

**Objective** In patients presenting with acute Type A aortic dissections (ATADs), the authors sought to evaluate whether emergent aortic operations performed by cardiac surgeons with different level of aortic surgery experience can impact perioperative outcomes and survival.

**Methods** A single-center review of 102 patients who underwent aortic surgeries for ATAD was conducted. The cohort was divided into those operated on by aortic specialists (AS:3 surgeons) and non-AS (5 surgeons). Multivariable logistic regression and Cox proportional hazard models were fitted to evaluate associations between the surgeon experience, perioperative outcomes, and survival, respectively.

**Results** Of 102 patients, 60 were operated on by AS and 42 were operated on by non-AS. Overall 30-day mortality was 11 (10.8%) with 4 (6.6%) perioperative deaths in the AS group and 7 (16.6%) among the non-AS group ( *p* = 0.2). AS performed a significantly higher number of root replacement procedures (41.6% vs. 23.8%, respectively, *p* = 0.049) and employed more frequent adjunct cerebral perfusion during circulatory arrest ( *p* = 0.003). Survival analysis indicated AS status was an independent predictor of improved 2-year survival (hazard ratio: 0.37, 95% confidence interval: 0.15–0.92, *p* = 0.03).

**Conclusion** Operation by AS for ATAD was associated with reduced adjusted risk of 2-year mortality. This adds support for establishing thoracic aortic emergency call teams staffed by AS.

## Introduction

Acute Type A aortic dissection (ATAD) is a devastating vascular event with a high pre-hospital and perioperative mortality ranging from 16 to 28%, 1 2 with a 1 to 2% hourly mortality rate. 3 Surgical mortality varies among different hospitals according to institutional volume and surgeon experience. 4 5 Dissection events have been shown to peak during early morning (6 a.m.–12 p.m.) and late afternoon hours. 6 Consequently, the majority of ATAD patients present at an unconventional time of the day, having no prior diagnosis of aortic disease, and usually require emergent, complex aortic replacement by an on-call cardiac surgeon. The complexity of ATAD surgery further diverges from a standard ascending aortic procedure as coexisting complications (e.g., malperfusion syndrome) demand adaptation and appropriate improvisation.

Some centers have instituted specialized aortic surgery teams that manage all emergent aortic cases. 7 In the context of temporal improvement in outcomes of aortic surgery owing to technological advancements (e.g., use of better grafts, availability of Bioglue [Cryolife, Kennasaw, GA]), 8 a contemporary benchmark for aortic surgical outcomes incorporating surgeon experience is needed.

We aimed to evaluate perioperative and 2-year outcomes associated with ATAD surgery performed by aortic specialists (AS) compared with cardiac surgeons without extensive aortic surgery background (non-AS).

## Materials and Methods

We conducted a retrospective review of consecutive patients who were operated for ATAD between 2008 and 2017 at Yale New Haven Hospital (YNHH). Out of 223 patients presenting with acute aortic emergencies, 128 patients underwent ascending aortic surgical intervention and had a complete record of admission-intervention timings and outside medical reports (transferred patients). Further exclusions were made for patients who were found to have chronic, rather than acute, ascending aortic dissection (15 cases), traumatic aortic dissection (7 cases), or incomplete data (4 cases). The final cohort consisted of 102 ATAD cases that were surgically managed at YNHH. The cohort was divided into those operated by AS (3 surgeons) and non-AS (5 surgeons) cardiac surgeons. An aortic surgery specialist was defined as a cardiac surgeon who consistently performed ≥30 ascending aortic replacement cases annually during the most recent 3 years of practice. To risk stratify the patients, multiple baseline characteristics, presence of preoperative comorbidities, and complications due to dissection were included in the analysis. Hypotension was defined as a systolic blood pressure less than 90 mm Hg. Malperfusion was confirmed according to the clinical ruling of the emergency care provider and cardiac surgeon according to clinical symptoms and examination (e.g., pulselessness, pallor, sudden onset of abdominal pain, diarrhea, neurological deficits) at the YNHH. For patients who were directly transferred to the operating room from outside medical facilities, their diagnosis was confirmed through respective hospital record or by surgeon examination before the procedure was performed. The Yale Institutional Review Board approved this study (HIC # 2000021950) and individual patient consent was waived.

### Surgeon Experience

Surgeons in the AS and non-AS groups had a mean age of 52 ± 13.1 and 49 ± 2.5 years, respectively. The mean annual ascending aortic case volume for the AS group was 32.3 ± 2.1, contrasting to 6 ± 1.8 cases performed by the non-AS group. Surgeons in the AS group had a mean experience of 23.6 ± 10.3 years in cardiac surgery practice compared with 20.3 ± 6.0 years for the non-AS group.

### Outcome Definition

Perioperative outcomes evaluated were mortality and composite adverse events, which consisted of mortality and stroke. The primary outcome of interest in the time-dependent model postdischarge was death. The long-term follow-up and mortality data were acquired through patient follow-up records according to the methodology described by Peterss et al. 9

### Statistical Analysis

Categorical variables were compared using chi-square test to analyze significance of proportions in the AS and non-AS group. Continuous variables were represented as mean ± standard deviations and compared using two-tailed *t* -test. Multivariable logistic regression models were fitted to evaluate associations between the surgeon training experience and perioperative mortality. A Cox proportional hazard model was fitted to evaluate associations between the surgeon training experience and 2-year survival. Proportional hazard assumption was evaluated with log–log curve, and covariates not satisfying the assumption were stratified in the Cox model. Kaplan–Meier and log rank analysis was performed to compare 2-year mortality rates between the two groups. SAS software Version 9.4 (SAS Institute Inc., Cary, NC) was used for the analysis. Statistical significance was set at *p* < 0.05.

## Results

The study population consisted of 102 patients, an aggregate of 70 (68.9%) male and 32 (31.1%) female patients. The overall mean age of subjects at the time of surgery was 59.2 ± 14.3 years and did not differ significantly between the AS and non-AS groups ( Table 1 ). Out of 102 patients, 60 patients were operated by AS and 42 by non-AS. Preoperative characteristics ( Table 1 ) were similar between patients operated by AS and non-AS, except for a higher frequency of chronic obstructive pulmonary disease (COPD) in the non-AS group (26.2% vs. 6.7%, *p* = 0.009). Severe aortic insufficiency was demonstrated in 19.7% of AS and 33.3% of non-AS on the preoperative echocardiogram ( *p* = 0.12).

**Table 1:** Preoperative/baseline patient characteristics for the aortic specialist and non-aortic specialist group

Variables	Non-AS ( *n* = 42)	AS ( *n* = 60)	*p* -Value
Mean (SD) age, y	59.2 ± 14.6	59.2 ± 14.3	1.0
Male	30 (71.4%)	40 (66.7%)	0.7
*Race:*			
Caucasian	32 (76.2%)	40 (66.7%)	0.3
African-American	8 (19.1%)	11 (18.3%)	
Other	2 (4.8%)	9 (15.0%)	
Mean (SD) BMI (kg/m ^2^ )	30.4 ± 5.5	28.5 ± 6.4	0.13
Transfer from outside facility	22 (52.4%)	35 (58.3%)	0.7
Time from admission to CT (min)	113.72 ± 107.1	123 ± 186.09	0.77
Admission-incision interval (h)	5.68 ± 3.5	5.88 ± 4.7	0.81
*Baseline comorbidities:*			
Hypertension	38 (90.5%)	49 (81.7%)	0.3
Mean (SD) eGFR	39.0 ± 33.2	37.3 ± 31.2	0.8
COPD	11 (26.2%)	4 (6.7%)	**0.009**
Coronary artery disease	9 (21.4%)	5 (8.3%)	0.08
Previous PCI	1	0	1.0
Previous CABG	3	1	1.0
Medication only	5	4	1.0
History of stroke	4 (9.5%)	1 (1.7%)	0.2
Previous cardiac surgery	3 (7.1%)	2 (3.3%)	0.4
Prior AVR	0	1	1.0
Prior CABG	3	0	
Mean (SD) hematocrit (%)	37.5 ± 8.1	38.6 ± 5.9	0.5
Mean (SD) platelets	222.2 ± 103.7	205.9 ± 75.8	0.4
*Dissection-related variables:*			
DeBakey Type I	35 (83.3%)	47 (78.3%)	0.6
Hypotension	5 (11.9%)	5 (8.3%)	0.7
Bicuspid aortic valve	3 (7.1%)	3 (5.0%)	0.7
Aortic arch involvement	39 (92.9%)	51 (85.0%)	0.4
Abdominal aorta involvement	25 (59.5%)	34 (56.7%)	0.8
Iliac artery involvement	17 (40.5%)	23 (38.3%)	0.8
Bovine arch	5 (11.9%)	10 (16.7%)	0.6
Malperfusion present	12 (28.6%)	17 (28.3%)	1.0
Cardiac tamponade	9 (21.4%)	17 (28.3%)	0.5
Aortic valve involvement	12 (28.5%)	12 (20%)	0.29
Aortic root involvement	28 (66.6%)	34 (56.6%)	0.26

Abbreviations: AS, aortic specialist; AVR, aortic valve replacement; BMI, body mass index; CABG, coronary artery bypass grafting; COPD, chronic obstructive pulmonary disease; CT, computed tomography; eGFR, estimated glomerular filtration rate; PCI, percutaneous coronary intervention; SD, standard deviation.

Note: Boldfaced values represent *p* < 0.05.

The AS group performed more root replacements (41.6% vs. 23.8%, respectively, *p* = 0.049) with higher rates of composite (Bentall procedure) root replacements than the non-AS group (Bentall procedure, 26.7% vs. 2.4%, *p* < 0.001). The AS group also employed frequent adjunct cerebral perfusion in cases with circulatory arrest (58.8%) compared with the non-AS group (14.3%) ( *p* < 0.001) ( Table 2 ).

**Table 2:** Operative management performed by the aortic specialist and non-aortic specialist group

Variables	Non-AS ( *n* = 42)	AS ( *n* = 60)	*p* -Value
Root replacement Bentall is a different variable	10 (23.8%) 1 (2.4%)	25 (41.6%) 16 (26.7%)	**0.049** **< 0.01**
Valve-sparing	0	3	0.3
Hemiarch replacement	37 (88.1%)	50 (83.3%)	0.6
Total arch replacement	1 (2.4%)	6 (10.0%)	0.2
Descending procedure	0 (0%)	4 (6.7%)	0.1
Concomitant CABG	4 (9.5%)	3 (5.0%)	0.4
DHCA use	42 (100%)	59 (98.3%)	1.0
Mean (SD) DHCA time, min	27.5 ± 12.5	28.9 ± 13.0	0.6
Adjunct cerebral perfusion	6 (14.3%)	39 (58.8%)	**< 0.001**
Antegrade	1	14	
Retrograde	5	25	
Mean (SD) Cx time, min	103.7 ± 45.2	107.0 ± 40.7	0.7
Mean (SD) CPB time, min	195.6 ± 53.5	185.8 ± 46.6	0.3

Abbreviations: AS, aortic specialist; CABG, coronary artery bypass grafting; CPB, cardiopulmonary bypass; Cx, aortic cross-clamp; DHCA, deep hypothermic circulatory arrest; SD, standard deviation.

Note: Boldfaced values represent *p* < 0.05.

In the entire cohort, mortality within 30 days of procedure occurred in 11 patients (10.8%), with 4 (6.6%) deaths in the AS group and 7 (16.6%) among the non-AS group ( *p* = 0.20). Logistic regression adjusting for 16 preoperative variables demonstrated that AS-status was not significantly associated with any difference in 30-day mortality (odds ratio [OR]: 0.32, 95% confidence interval [CI]: 0.07–1.30, *p* = 0.11). Four (9.5%) patients in the non-AS group and 7 (11.7%) patients in the AS group suffered from postoperative stroke ( *p* = 1.0). Improving renal function (estimated glomerular filtration rate) was protective of 30-day mortality (OR: 0.95, 95% CI: 0.91–0.99, *p* = 0.03) and concomitant coronary artery bypass grafting operation was associated with increased risk of postoperative early mortality (OR: 8.62, 95% CI: 1.23–60.53, *p* = 0.03).

A multivariate model assessing perioperative composite adverse events (30-day death and stroke) did not show significant association with surgeon specialty. The presence of preoperative malperfusion (OR: 3.19, 95% CI: 1.10–9.24, *p* = 0.03) and kidney injury (each unit increase in eGFR [OR: 0.97, 95% CI: 0.94–0.99, *p* = 0.02]) were associated with increased risk of perioperative composite adverse event.

Overall early postoperative outcomes ( Table 3 ) were comparable between the two groups. Median follow-up duration of patients in the non-AS and AS group was 20.1 (interquartile [IQ] range: 1.3–58.2) and 34.9 (IQ range: 5–58.2) months, respectively. The 6-month and 1-year survival for the AS group was 93.3 ± 3.3% and 88.7 ± 4.4%, respectively. For the non-AS group, 6-month and 1-year survival rate was 78.1 ± 6.5% and 78.1 ± 6.5%, respectively.

**Table 3:** Postoperative outcomes defined according to the aortic specialist and non-aortic specialist group

Variables	Non-AS ( *n* = 42)	AS ( *n* = 60)	*p* -Value
30-d mortality	7 (16.7%)	4 (6.7%)	0.2
Death < 24 h of surgery	0 (0%)	1 (1.7%)	1.0
Revision for bleeding	7 (16.7%)	8 (13.3%)	0.8
Stroke	4 (9.5%)	7 (11.7%)	1.0
Dialysis need	1 (2.3%)	1 (1.7%)	1.0
Mechanical ventilation > 48 h	17 (40.5%)	18 (30.0%)	0.3
Sepsis	3 (7.1%)	2 (3.3%)	0.4
Late aortic reoperation	4 (9.5%)	5 (8.3%)	1.0
Root/arch reoperation	4 (9.5%)	2 (3.3%)	0.22
Descending reoperation	0	3	−
Cause of death:			
Cardiogenic shock	4 (9.5%)	0 (0%)	0.03
Heart failure	1 (2.3%)	1 (1.7%)	
Malperfusion	0	1 (1.7%)	1
Multiorgan failure	2 (4.7%)	0	0.16
Stroke	0 (0%)	2 (3.3%)	

Abbreviation: AS, aortic specialist.

**Fig. 1:**
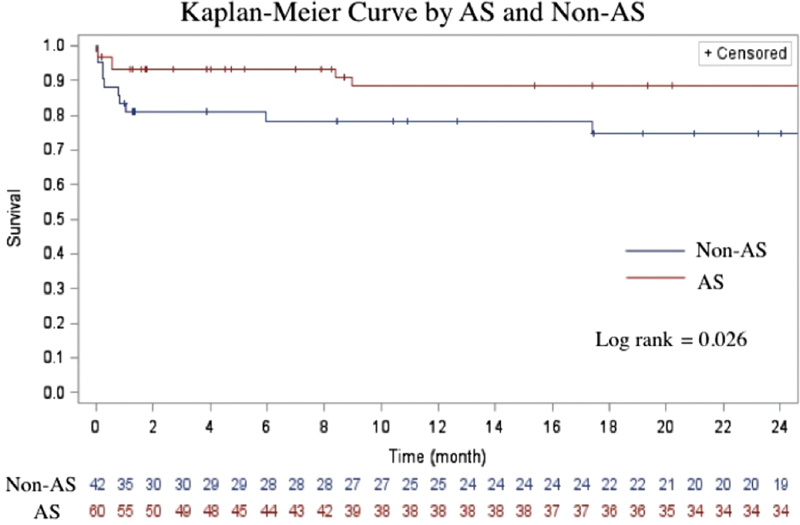
Kaplan–Meier curve demonstrating difference in 2-year survival among patients operated on by the aortic specialist (AS) group compared with the non-AS group ( *p* = 0.026).

Log rank analysis revealed a statistically significantly superior 2-year survival associated with surgery by the AS group ( *p* = 0.026) ( Fig. 1 ). Cox proportional hazard analysis also demonstrated AS-status as an independent predictor of improved survival at 2 years (hazard ratio: 0.37, 95% CI: 0.15–0.92, *p* = 0.03).

## Discussion

The overall 30-day mortality for ATAD in this study was 10.8% with 6.7% perioperative deaths in the group operated by the AS group. The stroke rate was comparable in the AS and non-AS groups even though the AS group adopted a higher rate of adjunct neuroprotective perfusion. Literature on cerebral protection is variable and adjunct perfusion may not be superior than isolated deep hypothermic circulatory arrest in ATAD cases; however, this observation is beyond the scope of this article. Concisely, patients operated on by the AS group had a significantly lower adjusted risk of 2-year mortality.

Recently, Bashir et al demonstrated improved outcomes of aortic surgery for ATAD associated with higher volume aortic surgeons based on the national U.K. data. 10 Their study demonstrated that surgeons performing on average ≥ 4 cases/year had lower in-hospital mortality than surgeons performing < 4 cases annually (12.6 and 19.3%, respectively, *p* = 0.015). 10 Similarly, Andersen et al 7 demonstrated a considerable decrease in perioperative mortality by around 25% after initiation of an exclusive aortic team in a single-center setting. This program restricted all thoracic aortic surgery to be performed by cardiac surgeons specialized in aortic surgery, leading to an improvement in operative mortality. 7 Another study looking at the volume outcome relations in “all-comer” (elective + emergent) ascending aorta/arch surgery highlighted lower midterm mortality risk for patients operated on by a high volume operator (HR: 0.67, 95% CI: 0.51–0.88). 11 Analysis of the national (U.S.) patient population by Hughes et al 12 revealed that patients operated at high volume aortic centers (30–100 cases/year) have considerably lower adjusted risk of mortality than low volume centers (< 6 cases/year) for elective ascending aortic surgery (OR: 0.42, 95% CI: 0.31–0.58). Notwithstanding this correlation (for all aortic surgery cases) of short-term (30-day) mortality with surgeon status, some results were inconsistent for emergent procedures and need further exploration in this specific group. 11 Increasing evidence supports experience–outcome relationships in high-risk operations, and this relationship is also expected to be reflected in emergent aortic surgery. 7 10 12

A potential solution advocated by some centers is for referral of all aortic cases to centers specialized for aortic surgery. Volume centralization is expected to improve outcomes but this may be difficult given the emergent nature of aortic dissection and difficulty in transferring/triaging these patients in a timely fashion. Case specialization within a given center may be a feasible alternative that improves the operative outcomes. We have implemented and also advocated the development of a designated “on-call aortic surgery” team. This involves an exclusive on-call AS in addition to the regular cardiac surgery attending staff. A protocol declaring the precise algorithm for rapid engagement of various additional members (nurses, physician assistants, perfusionists) on the team is also required for an efficient coordination. Our own protocol has proven to be very efficient for rapid triage and management of emergent aortic cases (dissection and rupture). 13

Although there were no major differences in the baseline characteristics or preoperative comorbid conditions between the two groups, there were some striking distinctions in the way these patients were managed. The AS group performed a significantly higher number of Bentall procedures than the non-AS group and also made relatively liberal use of adjunct cerebral perfusion. These choices could be partly explained in terms of greater surgeon comfort to adopt a more complex technique according to experience.

It was beyond the scope of this article to measure the exact factors leading to improved outcomes in the AS group. However, the results from this study argue for establishing thoracic aortic emergency on-call teams staffed by AS. This can be an efficient approach to manage all ATAD cases optimally; however, this methodology may only be practical at large centers that can afford a focused aortic team.

### Limitations

This study harbors limitations inherent to the single-center and retrospective design. The cohort size is relatively small due to the stringent exclusion criteria, and this likely limited a robust multivariable adjustment in the models, although statistical significance was reached in several of the variables outlined above. Selectivity of cases by each surgeon category could not be assessed, as the cohort consisted of only those who underwent the operation, although this single-center setting likely limited this heterogeneity. Furthermore, higher rates of baseline COPD and aortic insufficiency in the non-AS group likely prejudiced this sample for a dismal outcome.

## Conclusion

AS status of surgeons performing ATAD surgery defined improved 2-year survival for patients presenting with acute thoracic aortic emergencies. This argues for aortic surgery centers to form dedicated thoracic aortic emergency teams so that surgeons with aortic expertise are available to manage emergent cases at all times.
